# Analysis of retinal sublayer thicknesses and rates of change in *ABCA4*-associated Stargardt disease

**DOI:** 10.1038/s41598-020-73645-5

**Published:** 2020-10-06

**Authors:** S. Scott Whitmore, Christopher R. Fortenbach, Justine L. Cheng, Adam P. DeLuca, D. Brice Critser, Elizabeth L. Geary, Jeremy M. Hoffmann, Edwin M. Stone, Ian C. Han

**Affiliations:** Department of Ophthalmology and Visual Sciences, Carver College of Medicine, University of Iowa Institute for Vision Research, 200 Hawkins Drive, PFP 11196K, Iowa City, IA 52242 USA

**Keywords:** Clinical genetics, Biomarkers, Medical imaging, Clinical genetics, Translational research, Eye diseases

## Abstract

Stargardt disease, the most common inherited macular dystrophy, is characterized by vision loss due to central retinal atrophy. Although clinical trials for Stargardt are currently underway, the disease is typically slowly progressive, and objective, imaging-based biomarkers are critically needed. In this retrospective, observational study, we characterize the thicknesses of individual retinal sublayers by macular optical coherence tomography (OCT) in a large cohort of patients with molecularly-confirmed, *ABCA4*-associated Stargardt disease (STGD1) relative to normal controls. Automated segmentation of retinal sublayers was performed with manual correction as needed, and thicknesses in various macular regions were compared using mixed effects models. Relative to controls (42 eyes, 40 patients), STGD1 patients (107 eyes, 63 patients) had slight thickening of the nerve fiber layer and retinal pigment epithelium-Bruch’s membrane, with thinning in other sublayers, especially the outer nuclear layer (ONL) (p < 0.0015). When comparing the rate of retinal sublayer thickness change over time (mean follow-up 3.9 years for STGD1, 2.5 years for controls), STGD1 retinas thinned faster than controls in the outer retina (ONL to photoreceptor outer segments). OCT-based retinal sublayer thickness measurements are feasible in STGD1 patients and may provide objective measures of disease progression or treatment response.

## Introduction

Stargardt disease (Stargardt disease-1, STGD1; MIM #248200) is the most common cause of inherited macular dystrophy, with an estimated frequency of 1 in 10,000^1,2^. STGD1 is caused by mutations in *ABCA4* (ATP-binding cassette, sub-family A, member 4; OMIM #601691) which encodes a transmembrane transporter protein responsible for preventing the toxic accumulation of bisretinoids in the retinal pigment epithelium (RPE) that can lead to photoreceptor loss^3–7^. Clinically, the disease is characterized by yellow, fleck-like subretinal deposits, predominantly in the macula, over which there may be outer retinal loss. However, there is substantial heterogeneity with regard to age of onset, degree of vision loss, rate of progression, and extent of retinal involvement^8–10^.

Although the clinical features of STGD1 are well-described, debate remains regarding the exact sequence of tissue loss in this disease (e.g., whether RPE loss precedes photoreceptor loss, or vice versa). Most imaging-based studies of progression in STGD1 have assessed the area of RPE loss, which is readily measured using en face fundus autofluorescence (FAF) imaging^9,11–15^. However, recent studies using optical coherence tomography (OCT) have suggested that photoreceptor loss may actually precede RPE loss^16,17^. Longitudinal, en face evaluations of ellipsoid zone (EZ) area have shown that rates of photoreceptor and RPE loss are either similar or higher in photoreceptors, with a greater ratio of area loss in photoreceptors than RPE^17–19^. Relatively few studies to date have quantified the degree of OCT-based retinal thinning in STGD1 or attempted to assess the rate of retinal thickness change over time^20,21^. Moreover, although paradoxical thickening of the inner nuclear layer has been described^22^, relatively little is known about changes within retinal layers other than the photoreceptors or RPE.

In this study, we evaluate retinal sublayer changes as measured by OCT in a large cohort of patients with molecularly-confirmed *ABCA4*-associated Stargardt disease. We compare retinal thicknesses of these sublayers to an age-similar group of normal controls. We then describe regional differences in retinal thickness change based on macular zones and quantify the rate of change for various retinal sublayers over time.

## Methods

### Participants

**Figure 1:**
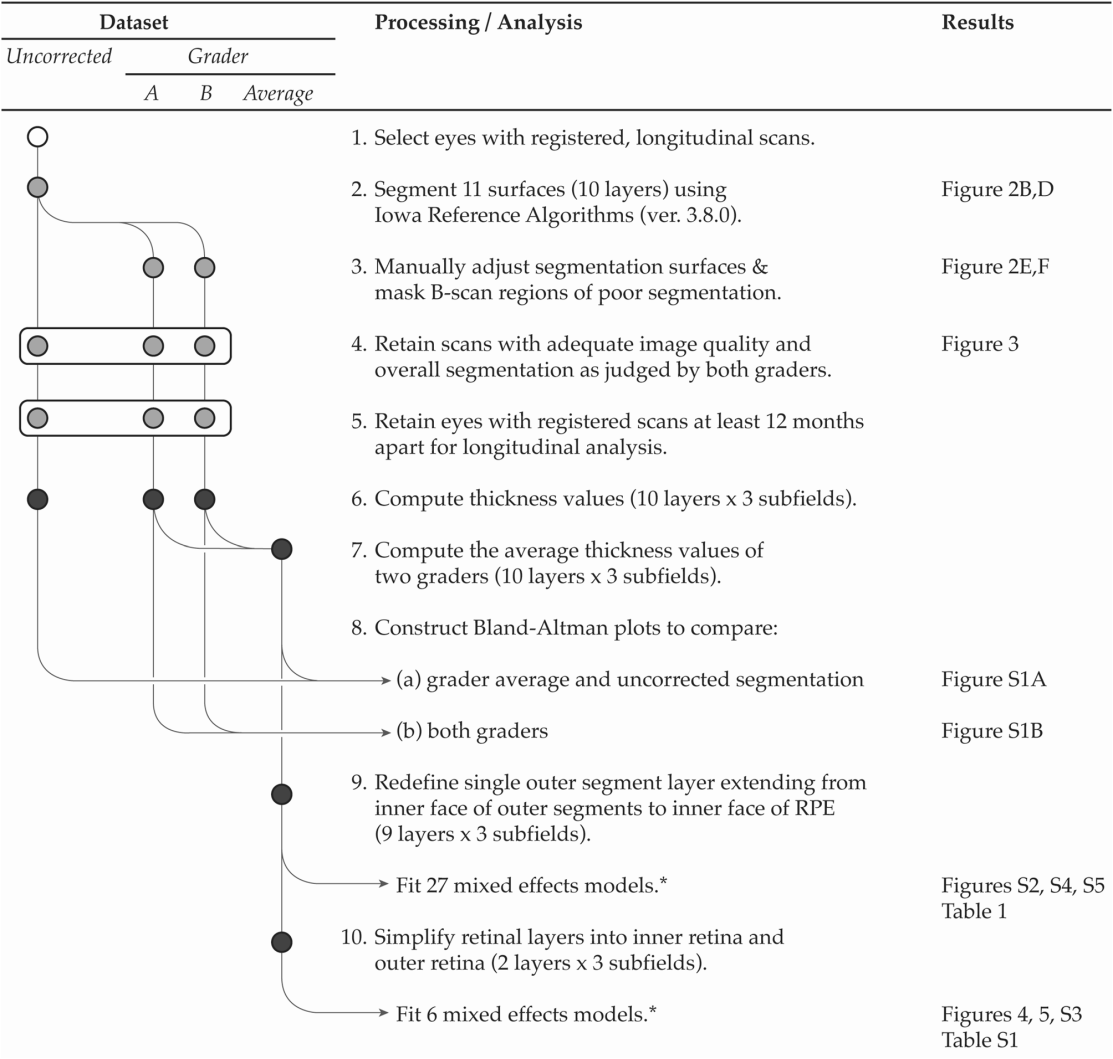
Overview of data processing and analysis. Shaded circles indicate data type (volume scans [white]; segmentation surfaces [light gray]; sublayer by subfield thicknesses [black]). The asterisk (*) indicates that a Bonferroni correction was applied for fitting 27 separate models (9 layers × 3 subfields) for individual sublayer analyses, and 6 separate models for combined sublayer analyses (“inner retina” and “outer retina” × 3 subfields). RPE = retinal pigment epithelium. “S” refers to Supplemental material. Designed in Adobe Illustrator (ver. 24.0.3; https://www.adobe.com/products/illustrator.html).

This is a retrospective, observational study evaluating OCT volume scans from a previously-published cohort of patients with molecularly-confirmed STGD1 seen at the University of Iowa from January 2010 to June 2016^2^. The study adhered to the tenets of the Declaration of Helsinki and was conducted in accordance with regulations set forth by the Health Insurance Portability and Accountability Act. Institutional Review Board approval for the study was obtained from the Human Subjects Committee of the University of Iowa, and informed consent was obtained for study participation. For participants under the age of 18 years, informed consent was obtained from a parent and/or legal guardian. Demographic data including gender and age at each visit were recorded for each patient. Best-corrected Snellen visual acuity was recorded for each eye at each study date and converted to LogMAR measurements as previously described^23,24^. Data processing, image analysis, and statistical comparisons are summarized in Fig. 1, and further described in detail below.

### OCT image acquisition and segmentation

Optical coherence tomography (Spectralis OCT; Heidelberg Engineering, Heidelberg, Germany) macular volume scans from patients with STGD1 were included in the study. These scans were obtained during the course of routine clinical care and included fovea-centered macular volume scan protocols that ranged from 20 degrees × 20 degrees to 30 × 20 degrees, with 512, 768 or 1024 (horizontal) × 496 (vertical) pixel density, and comprised of 19, 25, 31, 37, 47, or 49 B-scans. All scans taken in follow-up were acquired using TruTrack Active Eye Tracking with registration to prior scans.

For analysis of longitudinal data, only eyes with registered volume scans separated by at least 12 months were selected. When multiple registered scans were available, only the earliest and latest scans were used. We then segmented these scans using the Iowa Reference Algorithms (version 3.8.0; Retinal Image Analysis Lab, Iowa Institute for Biomedical Imaging, Iowa City, IA). These automated algorithms identify the foveal pit and segment the retina into 11 surfaces from the internal limiting membrane to the RPE with reliability similar to manual segmentation by expert human graders in normal eyes^25,26^.

**Figure 2:**
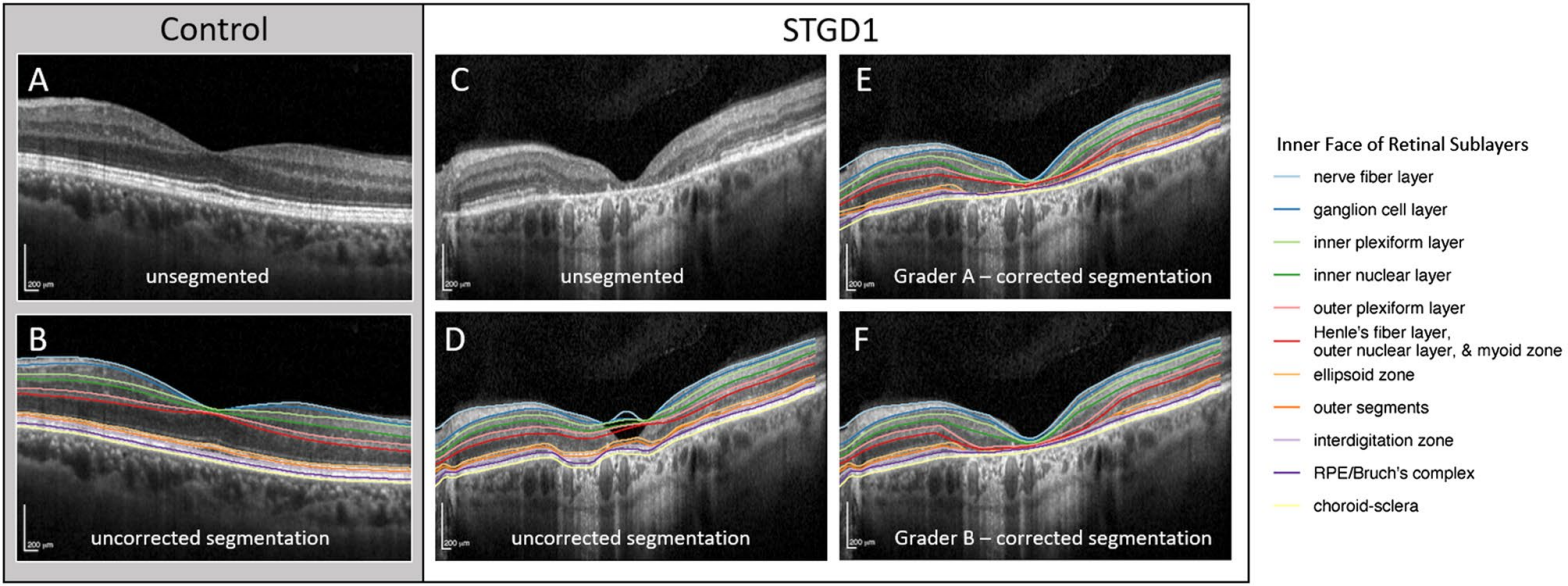
Example of corrected and uncorrected segmentation from transfoveal B-scans in a normal eye and in an eye with Stargardt disease. Unsegmented (**A**) and uncorrected segmented (**B**), B-scan from a 45-year-old control patient with no retina disease. (**C**–**F**) Central B-scan from a 55-year-old patient with Stargardt disease (STGD1), showing manual correction (**E**,**F**) of algorithm errors (**D**). Color-coded surfaces correspond to the inner face of retinal sublayers noted in the legend (right). Generated from data using R (ver. 2.6.3; https://www.r-project.org/).

All automatically segmented volume scans were then reviewed for accurate segmentation and foveal centration by two independent, ophthalmologist graders (C.R.F., J.L.C.), and scans with quality insufficient for segmentation (e.g., due to media opacity or severe disease such that retinal sublayers were indistinguishable) were excluded. For the remainder of the volume scans deemed acceptable, graders then manually corrected errors in segmentation surfaces and fovea location using the editing functions within the Iowa Reference Algorithms software (Fig. 2), which allows graders to manually adjust each retinal surface per individual B-scan and automatically propagates these changes to adjacent B-scans.

### Retinal thickness measurements of individual and combined retinal sublayers

We then computed retinal sublayer thicknesses per scan in three Early Treatment Diabetic Retinopathy (ETDRS) subfields (“center” 1-mm diameter circle centered on the fovea; “inner ring” of 3-mm diameter circle concentric to center; and “outer ring” of 6-mm diameter circle concentric to the inner ring) for each grader and in the uncorrected segmentations. Next, we averaged the corrected thickness values for each layer and subfield. We constructed Bland–Altman plots to assess agreement of thickness values (a) between grader average and uncorrected segmentation and (b) between both graders (Supplemental Fig. S1)^27^. To account for multiple measurements arising from the same patient and from the same eye, we adapted the method presented by Parker and coworkers for computing limits of agreement^28,29^. For each of the two comparisons, we first computed the difference between thickness measurements (either the uncorrected segmentation minus the grader average or Grader A minus Grader B). Next we fit the differences per patient (*id*) per *eye* and per *visit* to a linear mixed effects model, accounting for the correlation within patients and within eyes of a patient:

$$Differenc{e}_{id,eye,visit}={\mu }_{1}+{\alpha }_{1id}+{\beta }_{1eye}+{\epsilon }_{1id,eye,visit}$$

$${\alpha }_{1id}\sim N(0,{\sigma }_{1\alpha }^{2}), {\beta }_{1eye}\sim N(0,{\sigma }_{1\beta }^{2}), {\epsilon }_{1id,eye,visit}\sim N(0,{\sigma }_{1\epsilon }^{2})$$

where ${\mu }_{1}$ is the mean bias, ${\alpha }_{1id}$ is a random intercept for each patient, ${\beta }_{1eye}$ is a random intercept for each unique eye, and ${\epsilon }_{1id,eye,visit}$ is the residual for every patient, eye, and visit. We fit a second linear mixed effects model adjusting for the years since baseline visit:

$$Differenc{e}_{id,eye,age}={\mu }_{2}+{\gamma }_{2}\cdot year{s}_{id,eye,visit}+{\alpha }_{2id}+{\beta }_{2eye}+{\epsilon }_{2id,eye,visit}$$

$${\alpha }_{2id}\sim N(0,{\sigma }_{2\alpha }^{2}), {\beta }_{2eye}\sim N(0,{\sigma }_{2\beta }^{2}), {\epsilon }_{2id,eye,visit}\sim N(0,{\sigma }_{2\epsilon }^{2})$$

where ${\gamma }_{2}$ is the fixed effect years since baseline visit. The upper and lower limits of agreement were computed based on the unadjusted mean bias from the first equation and the variances of the random intercepts and the residual term from the second equation:

$${\mu }_{1}\pm 1.96\sqrt{{\sigma }_{2\alpha }^{2}+{\sigma }_{2\beta }^{2}+{\sigma }_{2\epsilon }^{2}}$$

We computed 95% confidence intervals around the mean bias, upper limit of agreement, and lower limit of agreement using a stratified bootstrap procedure. For each bootstrap ($n$ = 1999), we stratified the data by number of visits (OCTs) per patient, resampled the data with replacement at the patient-level within each stratum, fit both mixed effects models above, estimated the mean bias, and calculated the limits of agreement. We computed confidence interval boundaries by calculating the 2.5% and 97.5% quartiles. All models were fit using the lme function from the nlme package (ver. 3.1; [Pinheiro J, Bates D, DebRoy S, Sarkar D, R Core Team (2020). nlme: Linear and Nonlinear Mixed Effects Models. R package version 3.1-144, < URL:https://CRAN.R-project.org/package=nlme >]) for R (ver. 3.6.3; [R Core Team (2020). R: A language and environment for statistical computing. R Foundation for Statistical Computing, Vienna, Austria. URL https://www.R-project.org/]). We set the “control” parameter to be “lmeControl(opt = ‘optim’)”.

The grader average values for each of the 10 retinal sublayers were then computed. A single, combined sublayer for the outer segments (OS) was formed by taking the measurements from the inner face of the outer segments to the inner face of RPE/Bruch’s complex, resulting in a total of nine segmented retinal sublayers for subsequent analysis. For patients with available longitudinal imaging, the rate of change in retinal sublayer thicknesses (microns/year) was calculated using the initial and most recent follow-up scan. A subanalysis of these data were performed using three combined retinal layers: (1) inner retina [nerve fiber layer (NFL) to outer plexiform layer (OPL)]; (2) outer retina [outer nuclear layer (ONL) to outer segments (OS); and (3) RPE.

### Statistical analysis

For each eye from a STGD1 patient, we estimated (a) the thickness at baseline visit and (b) the rate of change over time for each sublayer and subfield. We then statistically compared these estimates to a cohort of age-similar normal eyes also with registered, longitudinal macular volume scans for one or more eyes, collected at least 12 months apart. To perform these comparisons, we modeled the data using mixed effects models^30^, which account for variation in the number of measurements per patient (one or two eyes) and for variation in follow-up duration. We performed modeling using the "nlme" package (ver. v.3.1-143; [Pinheiro J, Bates D, DebRoy S, Sarkar D, R Core Team (2019). nlme: Linear and Nonlinear Mixed Effects Models, < https://CRAN.R-project.org/package=nlme >]) for the R programming language [R Core Team (2019). R: A language and environment for statistical computing. R Foundation for Statistical Computing, Vienna, Austria. < https://www.R-project.org/ >].

We modeled thickness (in microns) as a function of years since baseline exam, group status (control or STGD1), and laterality of the eye (OD or OS). To evaluate how STGD1 status changes the rate of retinal thinning, we included an interaction term between years and group. We allowed baseline thickness and rate of change to vary by patient and by eye. Using the “lme” function from “nlme”, we specified the [main] model as `thickness ~ years + group + years:group’, specified the random effects as ‘~ 1 + years|id/eye’, and set the "control" parameter to ‘lmeControl (opt = 'optim', msMaxIter = 100)’. We evaluated this model for each combination of layers and subfields, for a total of 27 models (9 total layers [8 retinal sublayers + RPE] × 3 subfields).

In the longitudinal analysis, the rate of change for individual sublayer thicknesses was small for each intrinsically thin sublayer (e.g., outer plexiform layer, OPL). We therefore summed the thickness values for the inner retina (nerve fiber layer, NFL; through the OPL) and outer retina (outer nuclear layer, ONL; through outer segment, OS) and fit an additional 6 models (2 combined layers × 3 subfields). To account for multiple comparisons after fitting 33 independent models (11 total sublayers [9 retinal sublayers + “inner retina” + “outer retina”] × 3 subfields) to the dataset, we applied Bonferroni correction to determine statistical significance (p < 0.05 for one test is equivalent to p < 0.0015 after 33 tests). Error bars in supplemental figures represent the standard error of the mean for each group as estimated by the models.

### Meeting presentation

The results of this study were presented in part at the Annual Meeting of the Association for Research in Vision and Ophthalmology, Vancouver, Canada, in April 2019 [ARVO Abstract 2193].

## Results

**Figure 3:**
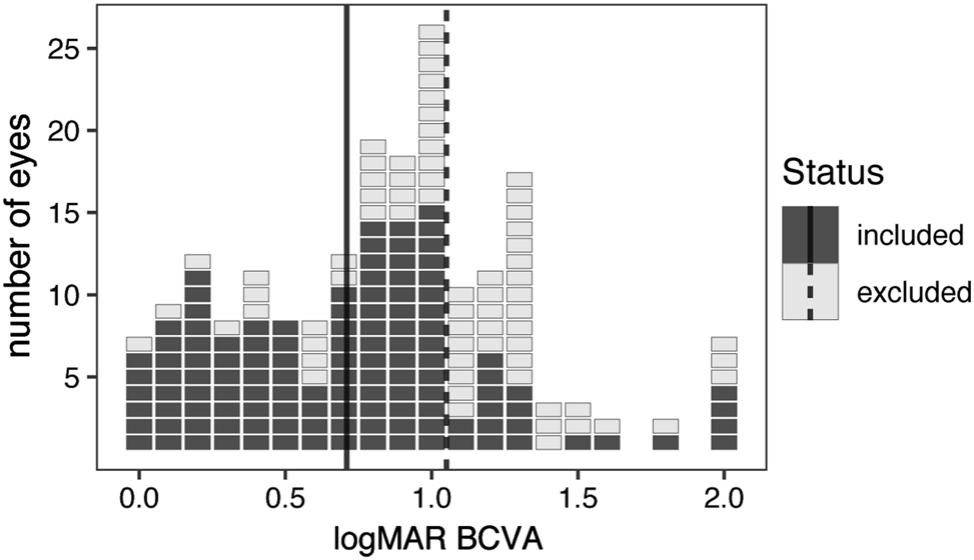
Visual acuity of eyes from Stargardt patients based on included or excluded status. Each rectangle represents one eye, with included versus excluded eyes shaded as per the figure legend. LogMAR visual acuities were derived from Snellen visual acuities as described in the “Methods” section. Mean acuities for included patients (solid vertical line) and excluded patients (dotted vertical line) are shown. Generated from data using R (ver. 2.6.3; https://www.r-project.org/).

A total of 162 Stargardt patients (64 male; 98 female; mean age at baseline = 35.6 years, range 6.3–82.3 years) were considered for the study. Of these, 127 patients had scans that could be reliably segmented; after removing eyes without longitudinal, registered scan pairs, our cohort for statistical analysis comprised 107 eyes from 63 patients (20 male, 43 female). All included scans for STGD1 patients required some manual modification of segmentation surfaces. For the STGD1 group, included patients were younger at baseline (mean age = 29.5 years, range 6.3–69.9 years) than excluded STGD1 patients (mean age 38.7 years, range 9.1–82.3 years), and mean visual acuity was better for included eyes (logMAR 0.7) than those we excluded (logMAR 1.1) (Fig. 3). An additional 40 individuals (20 male, 20 female; mean age at baseline = 46.8 years, range 14.1–78.9 years) without known ocular disease were included as controls. The mean interval of follow-up was 3.9 years (median 3.6, range 1.2–8.2) for STGD1 patients, and 2.5 years (median 2.3, range 1.0–6.5) for controls.

Examples of automated segmentation for a normal control eye, as well as correction by two graders for an ABCA4 eye, are shown in Fig. 2. The impact of manual correction varied depending on the ETDRS subfield analyzed, with the center subfield being more prone to algorithm error (e.g., Fig. 2D). The automated segmentation algorithm tended to underestimate the inner retinal layers (i.e., negative mean bias from the GCL through the INL) and to overestimate outer retinal sublayer thickness (i.e., positive mean bias in the ONL through the RPE) relative to manual corrections by the graders (e.g., Fig. 2D–F; Supplemental Fig. S1). Agreement between the two ophthalmologist graders was very good for all sublayers and subfields, and there was less observed measurement bias between the two graders than between the grader average and uncorrected segmentation. This decrease in bias after grader segmentation correction was most apparent for ONL measurements in the center subfield (Supplemental Fig. S1).

**Figure 4:**
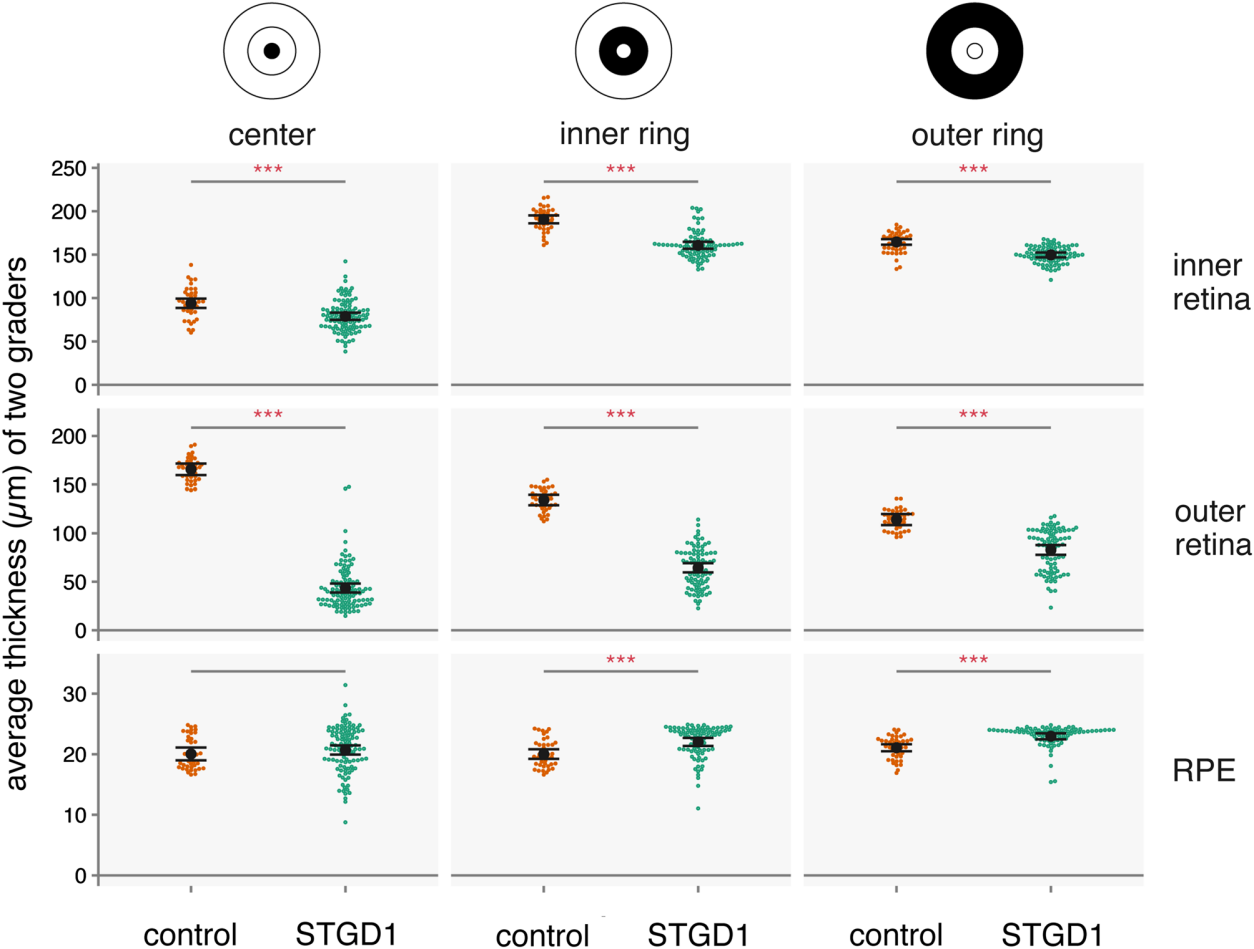
Average combined retinal layer thickness values at baseline for Stargardt versus normal controls. Each point represents the thickness value in one eye (orange = normal control; green = STGD1). The magnitude of p-values for each estimate are indicated by the number of asterisks (***p < 0.001; **p < 0.01; *p < 0.05). Red indicates p < 0.0015 (Bonferroni corrected equivalent of p < 0.05). Inner retina = nerve fiber layer to the outer plexiform layer. Outer retina = outer nuclear layer to the outer segments. RPE = retinal pigment epithelium. Generated from data using R (ver. 2.6.3; https://www.r-project.org/). ETDRS rings added in Adobe Illustrator (ver. 24.0.3; https://www.adobe.com/products/illustrator.html).

Baseline thicknesses were thinner in STGD1 patients than controls for all sublayers from the inner plexiform layer (IPL) through outer segments (OS) (Table 1; Supplemental Fig. S2). This was true for all subfields, but the center subfield manifested the greatest thinning and the outer ring manifested the least thinning (p < 0.0015 for all comparisons, except the outer rings of the ellipsoid zone and of the outer segment sublayers). Relative to controls, the greatest thinning for STGD1 patients was observed for the outer nuclear layer (ONL) for all subfields (center, − 79% thinner in STGD1 patients vs controls; inner ring, − 58%; outer ring, − 34%). Thickening in STGD1 patients compared to controls was observed in the NFL (center, inner ring) and the RPE (inner ring, outer ring) (p < 0.0015). Baseline retinal thickness measurement for the combined retinal layers (inner retina, outer retina) and RPE are shown in Fig. 4 and Supplemental Table S1.

**Table 1:** Baseline retinal thicknesses for nine retinal sublayers.

	Retinal sublayer	ETDRS subfield	Baseline retinal thickness (µm)				Difference (µm)	p-value
			Control group		STGD1 group			
			Mean ± SE	95% CI	Mean ± SE	95% CI		
Inner retina	Nerve fiber layer	Center	7.6 ± 0.7	6.1–9.1	11.1 ± 0.5	10.0–12.1	3.5	< 0.001
		Inner ring	28.7 ± 0.7	27.4–30.0	32.1 ± 0.6	31.0–33.3	3.4	< 0.001
		Outer ring	39.1 ± 0.7	37.7–40.4	39.0 ± 0.6	37.8–40.1	− 0.1	0.886
	Ganglion cell layer	Center	17.4 ± 1.1	15.3–19.5	17.7 ± 0.8	16.2–19.3	0.3	0.816
		Inner ring	52.5 ± 1.1	50.2–54.7	39.8 ± 1.0	37.9–41.7	− 12.6	< 0.001
		Outer ring	33.4 ± 0.6	32.3–34.5	28.4 ± 0.5	27.4–29.3	− 5.0	< 0.001
	Inner plexiform layer	Center	26.8 ± 0.9	25.0–28.5	19.7 ± 0.7	18.4–21.0	− 7.1	< 0.001
		Inner ring	39.5 ± 0.8	37.9–41.0	34.0 ± 0.7	32.7–35.3	− 5.5	< 0.001
		Outer ring	34.4 ± 0.6	33.3–35.5	29.8 ± 0.5	28.8–30.8	− 4.6	< 0.001
	Inner nuclear layer	Center	21.9 ± 1.0	19.9–23.8	17.3 ± 0.8	15.8–18.8	− 4.5	< 0.001
		Inner ring	40.4 ± 0.8	38.8–41.9	33.0 ± 0.7	31.7–34.4	− 7.3	< 0.001
		Outer ring	32.0 ± 0.5	31.1–32.9	29.1 ± 0.4	28.3–29.9	− 2.9	< 0.001
	Outer plexiform Layer	Center	20.1 ± 0.8	18.6–21.6	13.1 ± 0.6	11.9–14.2	− 7.1	< 0.001
		Inner ring	29.5 ± 0.6	28.2–30.7	21.6 ± 0.5	20.5–22.6	− 7.9	< 0.001
		Outer ring	25.5 ± 0.4	24.7–26.4	23.3 ± 0.3	22.6–24.0	− 2.2	< 0.001
Outer retina	Outer nuclear layer	Center	116.9 ± 2.1	112.8–121.1	24.1 ± 1.6	20.8–27.3	− 92.9	< 0.001
		Inner ring	91.4 ± 1.7	87.9–94.8	38.4 ± 1.5	35.4–41.3	− 53.0	< 0.001
		Outer ring	75.0 ± 1.9	71.2–78.8	49.3 ± 1.7	46.0–52.6	− 25.7	< 0.001
	Ellipsoid zone	Center	16.1 ± 0.6	14.9–17.3	7.4 ± 0.4	6.5–8.2	− 8.8	< 0.001
		Inner ring	14.8 ± 0.5	13.8–15.9	10.2 ± 0.5	9.3–11.1	− 4.6	< 0.001
		Outer ring	14.2 ± 0.5	13.3–15.1	12.4 ± 0.4	11.6–13.1	− 1.8	0.003
	Outer segment	Center	32.5 ± 1.3	30.1–35.0	12.1 ± 1.0	10.2–14.0	− 20.5	< 0.001
		Inner ring	28.1 ± 1.3	25.7–30.6	15.6 ± 1.1	13.5–17.8	− 12.5	< 0.001
		Outer ring	24.9 ± 1.1	22.6–27.1	21.0 ± 1.0	19.1–23.0	− 3.8	0.012
	Retinal pigment epithelium	Center	20.0 ± 0.5	19.0–21.1	20.7 ± 0.4	20.0–21.5	0.7	0.322
		Inner ring	20.0 ± 0.4	19.2–20.8	22.0 ± 0.3	21.4–22.7	2.0	< 0.001
		Outer ring	21.1 ± 0.3	20.5–21.7	23.0 ± 0.3	22.5–23.5	1.9	< 0.001

p-values of < 0.0015 are considered statistically-significant (Bonferroni corrected equivalent of 0.05).

**Figure 5:**
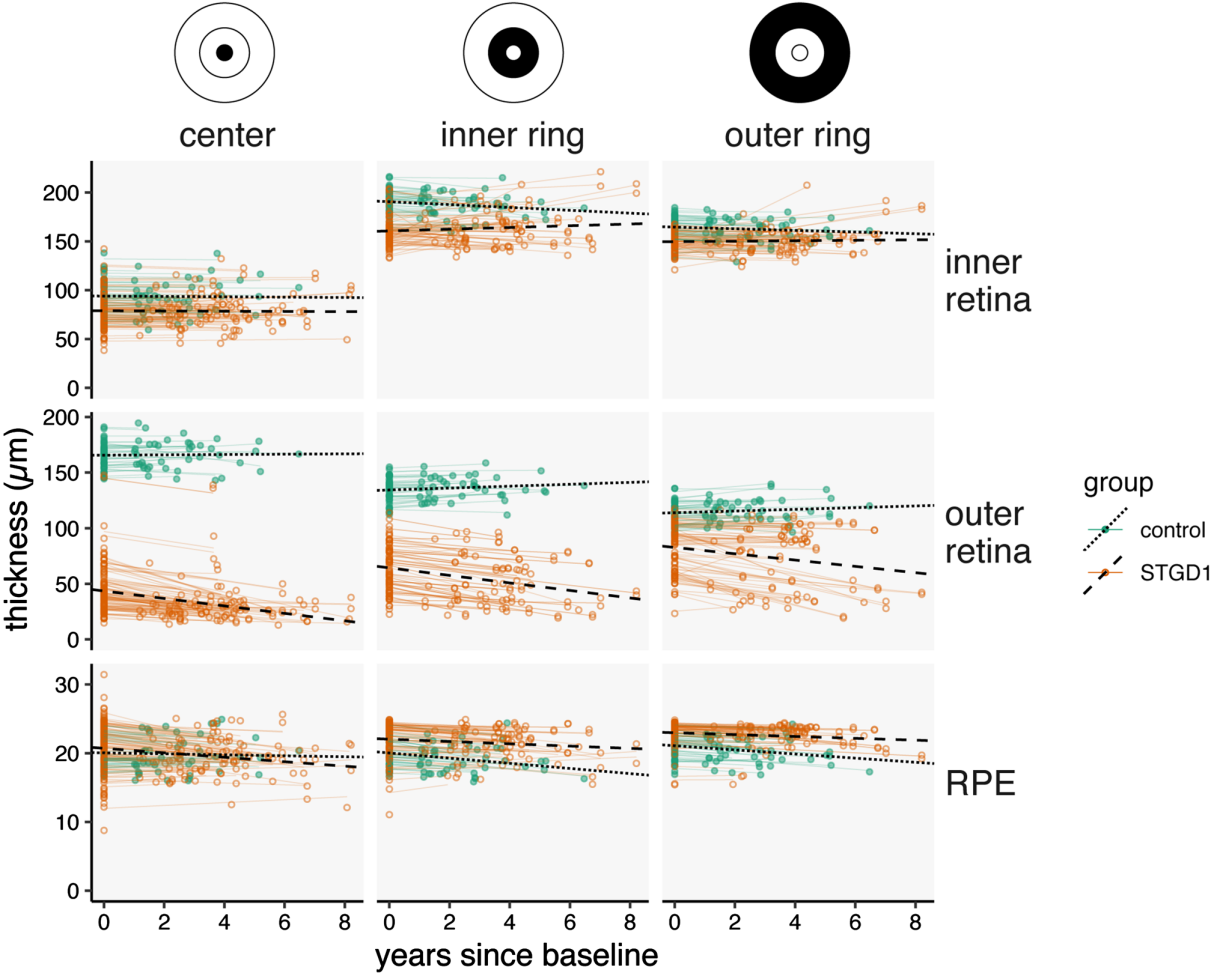
Change in combined retinal sublayer thickness per year by subfield in Stargardt eyes compared to control eyes. Each colored dot represents an average thickness of two graders per control (green) or Stargardt (STGD1) eye (orange). Thin colored lines indicate fits for individual eyes in the dataset. Thick black lines indicate fitted rates for control (dotted) or STGD1 eyes (dashed). Inner retina = nerve fiber layer to the outer plexiform layer. Outer retina = outer nuclear layer to the outer segments. RPE = retinal pigment epithelium. Generated from data using R (ver. 2.6.3; https://www.r-project.org/). ETDRS rings added in Adobe Illustrator (ver. 24.0.3; https://www.adobe.com/products/illustrator.html).

When evaluating the rate of thickness change in combined retinal sublayers (inner retina, outer retina) and the RPE, the outer retina (ONL to outer segments) of STGD1 patients thinned faster in all subfields than controls, who had relatively stable outer retina thicknesses (p < 0.0015; Supplemental Table S1). Individual patients, however, varied widely in the rate of change (Fig. 5, Supplemental Fig. S3). The inner retina and RPE in STGD1 patients had a slow rate of thickness change that did not differ statistically when compared to controls. Change in the retinal thickness of individual retinal sublayers is shown in Supplemental Figs. S4, S5.

## Discussion

OCT volume scans are routinely acquired as part of standard care for patients with macular disease, including Stargardt macular dystrophy. These volume scans contain a wealth of information regarding individual retinal sublayers that has not been thoroughly explored to date, in large part due to challenges in image segmentation and processing. For example, the Progression of Atrophy Secondary to Stargardt Disease (ProgStar) Study group previously demonstrated good reproducibility of total retinal, EZ, and ONL thickness measurements on SD-OCT in a small subset (30 patients) of patients as part of a prospective natural history study^31^. However, numerous challenges to segmentation were encountered, including difficulty identifying boundaries for pathologic areas of layer disruption and algorithm failures that required tedious (6–8 h per eye) manual segmentation^32^. Our results demonstrate that OCT-based analysis of individual retinal sublayer thicknesses is feasible for the majority of Stargardt patients seen in an inherited retinal disease clinic. In contrast to segmentation used in ProgStar, where manual correction of individual sublayers per each individual B-scan was performed, we used the Iowa Reference Algorithms, which take into account 3-dimensional data from adjacent B-scans in a volume scan to segment individual surfaces^25,26^. For all subfields, the uncorrected Iowa Reference Algorithms tended to overestimate the thickness of the outer retinal sublayers as compared to grader average, especially in the center subfield where disease was most severe (Supplemental Fig. S1). The inner ring and outer ring, however, had excellent grader agreement, suggesting that measurements in these subfields may be more reliable. This is in agreement with the ProgStar group’s conclusion that the inner ring is more reliable than the center subfield (outer ring analysis was not recommended due to incomplete volume scans in this subfield)^31^. In our study, manual correction of segmentation errors also greatly decreased measurement bias within the center subfield as well, and the impact of correction was most notable for the ONL.

As expected, measurements of the outer retinal layers were thinner for STGD1 patients relative to controls at baseline, but the degree of thinning relative to controls varied by zone, with the greatest thinning in the center subfield, followed by the inner and outer rings. Interestingly, the RPE layer was thicker than controls in the inner and outer rings. This finding is consistent with the known pathophysiology of the disease (accumulation of toxic bisretinoids within the RPE) and confirms prior OCT-based observation that the outer retina between Bruch’s membrane and the EZ is thickened^33^. In the inner and outer rings, the outer retinal sublayers (ONL to OS) are thin despite the RPE thickening in these regions, with the greatest difference in the ONL, consistent with prior OCT-based observations that loss of photoreceptor nuclei is observed first, followed by measurable loss of the photoreceptor inner/outer segments, and eventual RPE atrophy^21,34^.

Compared to outer retinal changes, relatively little is known regarding the inner retinal layers in patients with Stargardt disease. Interestingly, our results show that there is thickening of the NFL in the center subfield and inner ring relative to controls. Variable NFL thickening (about 15% of eyes) or thinning (about half of eyes) in Stargardt has been described^35^, but the etiology of this change is unclear, whether due to neuroinflammation, gliosis, or edematous thickening. We suspect that the NFL layer thickening may be an earlier manifestation of damming of axonal flow in this layer, akin to what has been described in other heritable photoreceptor degenerations. For example, in retinitis pigmentosa, decreased photoreceptor signal is thought to result in secondary alterations of axonal transport in the inner retina and optic nerve head drusen in some patients^36–38^. Thickening of the nerve fibers on histology and OCT can be seen even in the presence of GCL thinning and in the absence of gliosis^37–40^. By contrast, the GCL was thinner in Stargardt patients versus controls, particularly in the inner ring and outer ring. We suspect that this represents a secondary pruning of the retinal ganglion cell nuclei in response to photoreceptor loss, as is seen in many models of photoreceptor disease^41–43^. Interestingly, temporal optic nerve head pallor has been described in about 10% of Stargardt patients^44^, and the thinning of the GCL may explain the optic nerve head pallor noted on clinical examination. Huang and colleagues previously used OCT to follow Stargardt patients (n = 45) for 4 years and demonstrated paradoxical INL thickening, which they attributed to retinal remodeling in the context of photoreceptor disease^22^. In our study, baseline INL thickness was thinner in all subfields for STGD1 patients versus controls. However, some individual patients in the longitudinal group were noted to have INL thickening (Supplemental Fig. S4), and overall, there was a slight inner retinal thickening over time (0.89 microns/year, inner ring; 0.23 microns/year, outer ring). We also suspect that the INL thickening occurs as a retinal remodeling process earlier in the stage of disease before eventual thinning.

Detection of photoreceptor and RPE loss in STGD1 are method-dependent, and most of the previously-published work has used en face imaging using short-wavelength autofluorescence (SW-AF) or near-infrared autofluorescence (NIR-AF). These methods analyze disease progression by tracking area of involvement but require atrophic loss of RPE cells to measure a discrete, hypo-autofluorescent border, which can sometimes be difficult to distinguish (e.g., determination of disease borders on SW-AF can be challenging due to accumulation of hyper-autofluorescent material in photoreceptor cells)^32^. Studies using en face SW-AF or NIR-AF imaging have suggested that the rate of RPE loss correlates with EZ loss, particularly when the total area of abnormal autofluorescence (hypo- and hyper-autofluorescence) are considered using SW-AF; however, using NIR-AF, RPE atrophy appears to precede detectable photoreceptor loss^11,14,45–47^. When evaluating disease progression cross-sectionally using OCT, thinning of the ONL appears to the most prominent change, followed by measurable thinning of the photoreceptor inner/outer segments and RPE, as reported previously^21,34^. The overall rate of outer retina loss in our study was about 3 microns/year, and interestingly, the rate of loss was similar across all three ETDRS subfields. Unlike the outer retina, the RPE layer did not show significant thinning, likely because detection of thinning in this monolayer would require atrophic loss of the cells (i.e., regional loss of some but not all RPE cells may change the reflectivity of this layer on OCT but not necessarily the thickness). Because of the packing density of photoreceptor nuclei within the ONL, incremental loss of photoreceptor cells would result in detectable retinal thinning, even without complete loss of the retinal sublayer. Thus, monitoring OCT-based retinal thickness changes within the outer retinal sublayers (e.g., ONL and/or combined outer retina) may be a convenient way to detect mild changes before eventual cell loss and clinically visible atrophy. Further studies using cellular imaging (e.g., adaptive optics) may shed further light onto the sequence of photoreceptor and RPE cell loss in STGD1^48,49^.

Numerous clinical trials of gene therapy and stem cell therapy for Stargardt disease are currently underway, and inclusion of easily-acquired, objective measures of disease progression or treatment response remain critically needed^50–54^. Due to the relatively slow progression of STGD1 and the variability in foveal involvement, it is difficult to follow disease progression in clinical trials using best-corrected visual acuity^55^, and measurements are sometimes limited by ability to fixate and by changes in fixation location^56^. Other tests such as Goldmann visual fields are time-consuming and subjective, while electroretinography is also technically challenging in some patients with relatively low test repeatability, limiting its ability to detect small changes over time. Microperimetry with static perimetry may provide detailed functional testing in the macula but is also time-consuming, and Stargardt patients have less well-defined correlation between structure and function than patients with age-related macular degeneration^57–59^. Thus, combining structural and functional outcomes may be more sensitive to changes than a single mode of testing alone^60^. As above, our data demonstrate an overall slow rate of retinal change (e.g., 3 microns/year for outer retina thinning) in patients with STGD1. However, there were numerous individual outliers relative to the group (Fig. 5, Supplemental Fig. S4), and accounting for individual variability may be difficult in clinical trials, which are likely to involve relatively small numbers of patients given the relative rarity of the condition. These data suggest that retinal sublayer thickness measurements, though feasible, may not be ideal as a primary endpoint for clinical trials of short duration, but may be acceptable as a secondary outcome measure, or for studies with longer follow up (e.g., 10 years). In order to interpret retinal sublayer thickness change for use in clinical trials, combination with multimodal imaging and functional testing will be needed. Taking the volume of key anatomic layers (e.g. EZ, which has strong correlation to visual acuity) may be more robust than thickness alone^20^.

This study had many strengths, including the size of our study, which is the largest to date evaluating OCT-based findings in STGD1. In addition to clinical diagnosis by an inherited retinal disease expert, all included patients were molecularly-confirmed with two pathogenic variants in *ABCA4*. The study included scans from consecutive patients seen within an inherited eye disease clinic, and thus were less susceptible to selection bias from strict inclusion criteria as might be seen in a prospective clinical trial. Another strength of our study is the inclusion of a control group of eyes without ocular disease, allowing more informed interpretation of those changes that may be driven by *ABCA4*-related pathology versus other factors such as age. Although the control group was older (mean age 46.8 versus 29.5 years for STGD1 patients), the differences between the two groups are perhaps more convincing in this context, as retinal thicknesses typically decline with age^61,62^. We evaluated retinal thicknesses within standard ETDRS subfields, but future studies may evaluate the variations in foveal involvement and retinal thinning within different foveal zones. Due to the numerous layers analyzed in this study, we also did not include analysis of individual ETDRS subfields (i.e., nasal, superior, inferior, temporal) within the inner or outer rings. Because this study was designed to evaluate retinal thickness changes within various retinal sublayers, we did not evaluate the degree of reflectance (i.e., the texture) of each sublayer, but this may be the focus of future studies.

This study was limited by its retrospective nature, with inclusion of macular volume scans acquired during routine clinical care. Because of this, there may be small differences in the data if the scans were acquired in a more standardized setting of a prospective study, and we did not evaluate the impact of scan density on our results^63^. About a third of eyes were excluded due to poor image quality or advanced disease where the retinal laminations were indistinct, so as expected, the data are biased toward patients with milder disease. Retinal thickness measurements can be affected by axial length^64^, but we did not have axial length measurements available on most patients to examine its impact. To evaluate retinal thickness changes over time, we chose statistical models that assume a linear change in retinal thickness over time. While these models provide a reasonable representation of retinal thickness changes for this population over a relatively short period of time (e.g., several years, as in this study), they are less ideal for evaluating the likely non-linear progression of disease over the course of an individual patient’s lifetime. Most of the patients included in this study had a slow rate of retinal thinning over time. However, a few patients were noted to be outliers, and future study may evaluate factors influencing the rate of retinal thinning. Although all patients in our study had molecular-confirmation of STGD1, the analysis of genotype and its potential impact on these results was beyond the scope of this particular study.

To our knowledge, this is the largest study to date evaluating OCT-based retinal sublayer thickness measurements in STGD1. Compared to controls, STGD1 eyes possess a distinct pattern of both inner and outer retinal thinning compared to age-similar normal controls that can be readily-identified and measured on OCT. Longitudinal, OCT-based assessment of the rate of retinal sublayer change is also feasible, and these measurements may provide objective ways of monitoring disease progression or treatment response for clinical trials over time. The rate of outer retinal thinning in STGD1 is generally very slow, but significant individual variations exist. These individual differences relative to the population may help identify outliers of interest for future studies using induced pluripotent stem cell-derived cell-based disease modeling and genetic analysis for possible modifier genes, which may in turn provide further insight into Stargardt disease pathogenesis and its pathophysiologic mechanisms.

## Supplementary information

Supplementary Information.
