# Supplementary material for: Analysis of retinal sublayer thicknesses and rates of change in ABCA4-associated Stargardt disease
Source: Sci Rep. 2020 Oct 6;10:16576. doi: 10.1038/s41598-020-73645-5 (PMC7538899; doi:10.1038/s41598-020-73645-5)
Supplement: Supplementary file 1 — Supplementary Information. [file 41598_2020_73645_MOESM1_ESM.docx]

Supplementary Information

**Analysis of Retinal Sublayer Thicknesses and Rates of Change in *ABCA4*-Associated Stargardt Disease**

S. Scott Whitmore, PhD^1^; Christopher R. Fortenbach, MD, PhD^1^; Justine L. Cheng, MD^1^; Adam P. DeLuca, PhD^1^; D. Brice Critser, BS, CRA^1^; Elizabeth L. Geary, MS^1^; Jeremy M. Hoffmann, BS^1^; Edwin M. Stone, MD, PhD^1^; *Ian C. Han, MD^1^

**Author Affiliations:** ^1^ University of Iowa Institute for Vision Research, Department of Ophthalmology and Visual Sciences, Carver College of Medicine, Iowa City, IA 52242

**Corresponding Author:** Ian C. Han, MD; Department of Ophthalmology and Visual Sciences, University of Iowa Carver College of Medicine; 200 Hawkins Drive, PFP 11196K; Iowa City, IA 52242; email: [ian-han@uiowa.edu](mailto:ian-han@uiowa.edu)

**Supplemental Table 1.** Baseline retinal thicknesses and rates of thickness change for combined inner and outer retinal layers and retinal pigment epithelium in Stargardt (STGD1) patients versus normal controls.

|  |  | **Baseline Retinal Thickness (µm)** | | | |  |  | **Change in Thickness (µm/year)** | | | |  |  |
| --- | --- | --- | --- | --- | --- | --- | --- | --- | --- | --- | --- | --- | --- |
|  |  | **Control Group** | | **STGD1 Group** | |  |  | **Control Group** | | **STGD1 Group** | |  |  |
| **Retinal Layers** | **ETDRS Subfield** | **Mean ± SE** | **95% CI** | **Mean ± SE** | **95% CI** | **Difference (µm)** | **P-value** | **Mean ± SE** | **95% CI** | **Mean ± SE** | **95% CI** | **Difference (µm/year)** | **P-value** |
| **inner retina** | center | 94.0±2.8 | 88.6—99.4 | 78.9±2.1 | 74.8—83.1 | -15.0 | <0.001 | -0.18±0.72 | -1.60—1.24 | -0.11±0.35 | -0.80—0.57 | 0.07 | 0.931 |
|  | inner ring | 190.6±2.3 | 186.0—195.1 | 160.7±2.0 | 156.7—164.7 | -29.9 | <0.001 | -1.46±0.79 | -3.01—0.09 | 0.89±0.64 | -0.37—2.15 | 2.35 | 0.023 |
|  | outer ring | 164.6±1.7 | 161.4—167.9 | 149.7±1.4 | 146.9—152.5 | -14.9 | <0.001 | -0.85±0.52 | -1.87—0.17 | 0.23±0.42 | -0.59—1.06 | 1.08 | 0.108 |
| **outer retina** | center | 165.6±3.0 | 159.7—171.5 | 43.6±2.4 | 38.9—48.2 | -122.0 | <0.001 | 0.15±0.71 | -1.23—1.53 | -3.35±0.42 | -4.17—-2.54 | -3.5 | <0.001 |
|  | inner ring | 134.3±2.7 | 128.9—139.6 | 64.4±2.4 | 59.7—69.1 | -69.9 | <0.001 | 0.86±0.59 | -0.29—2.00 | -3.39±0.39 | -4.16—-2.62 | -4.24 | <0.001 |
|  | outer ring | 114.1±2.9 | 108.4—119.8 | 82.8±2.6 | 77.7—87.8 | -31.3 | <0.001 | 0.76±0.55 | -0.32—1.85 | -2.84±0.46 | -3.73—-1.95 | -3.6 | <0.001 |
| **RPE** | center | 20.0±0.5 | 19.0—21.1 | 20.7±0.4 | 20.0—21.5 | 0.7 | 0.322 | -0.07±0.16 | -0.38—0.24 | -0.32±0.08 | -0.48—-0.17 | -0.25 | 0.152 |
|  | inner ring | 20.0±0.4 | 19.2—20.8 | 22.0±0.3 | 21.4—22.7 | 2.0 | <0.001 | -0.37±0.11 | -0.59—-0.16 | -0.17±0.07 | -0.30—-0.04 | 0.21 | 0.105 |
|  | outer ring | 21.1±0.3 | 20.5—21.7 | 23.0±0.3 | 22.5—23.5 | 1.9 | <0.001 | -0.30±0.07 | -0.44—-0.16 | -0.13±0.05 | -0.22—-0.05 | 0.17 | 0.054 |

Inner retina = nerve fiber layer to the outer plexiform layer. Outer retina = outer nuclear layer to the outer segments. RPE = retinal pigment epithelium.  P-values of < 0.0015 are considered statistically-significant (Bonferroni corrected equivalent of p<0.05).

**Supplemental Figure 1.** Bland-Altman analysis of agreement between uncorrected and corrected segmentation for Stargardt patients by retinal sublayer and subfield.


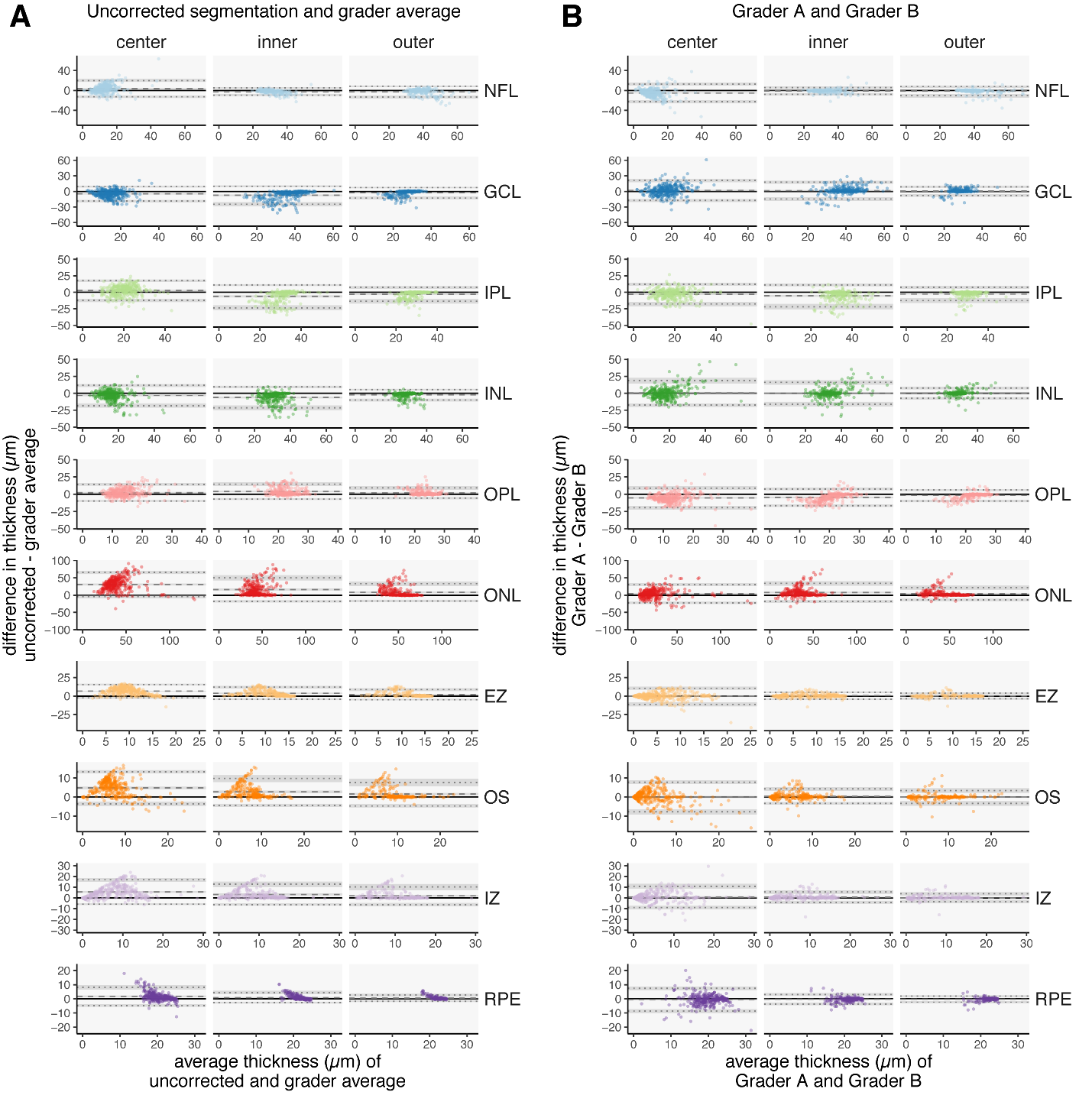


(A) Agreement between uncorrected and corrected (average of two, independent ophthalmologist graders) segmentation. (B) Agreement between ophthalmologist graders. Each point represents the retinal sublayer thickness value in one eye. Each point represents the retinal sublayer thickness value in one eye at one visit. The mean bias is displayed as the horizontal dotted line for each plot, with upper and lower dotted lines representing the 97.5% and 2.5% limits of agreement, respectively. 95% confidence intervals are displayed as gray bands. NFL = nerve fiber layer; GCL = ganglion cell layer; IPL = inner plexiform layer; INL = inner nuclear layer; OPL = outer plexiform layer; ONL = outer nuclear layer; EZ = ellipsoid zone; OS = outer segment; IZ = interdigitation zone; RPE = retinal pigment epithelium. Generated from data using R (ver. 2.6.3; https://www.r-project.org/). Plots combined and annotated in Adobe Illustrator (ver. 24.0.3; https://www.adobe.com/products/illustrator.html).


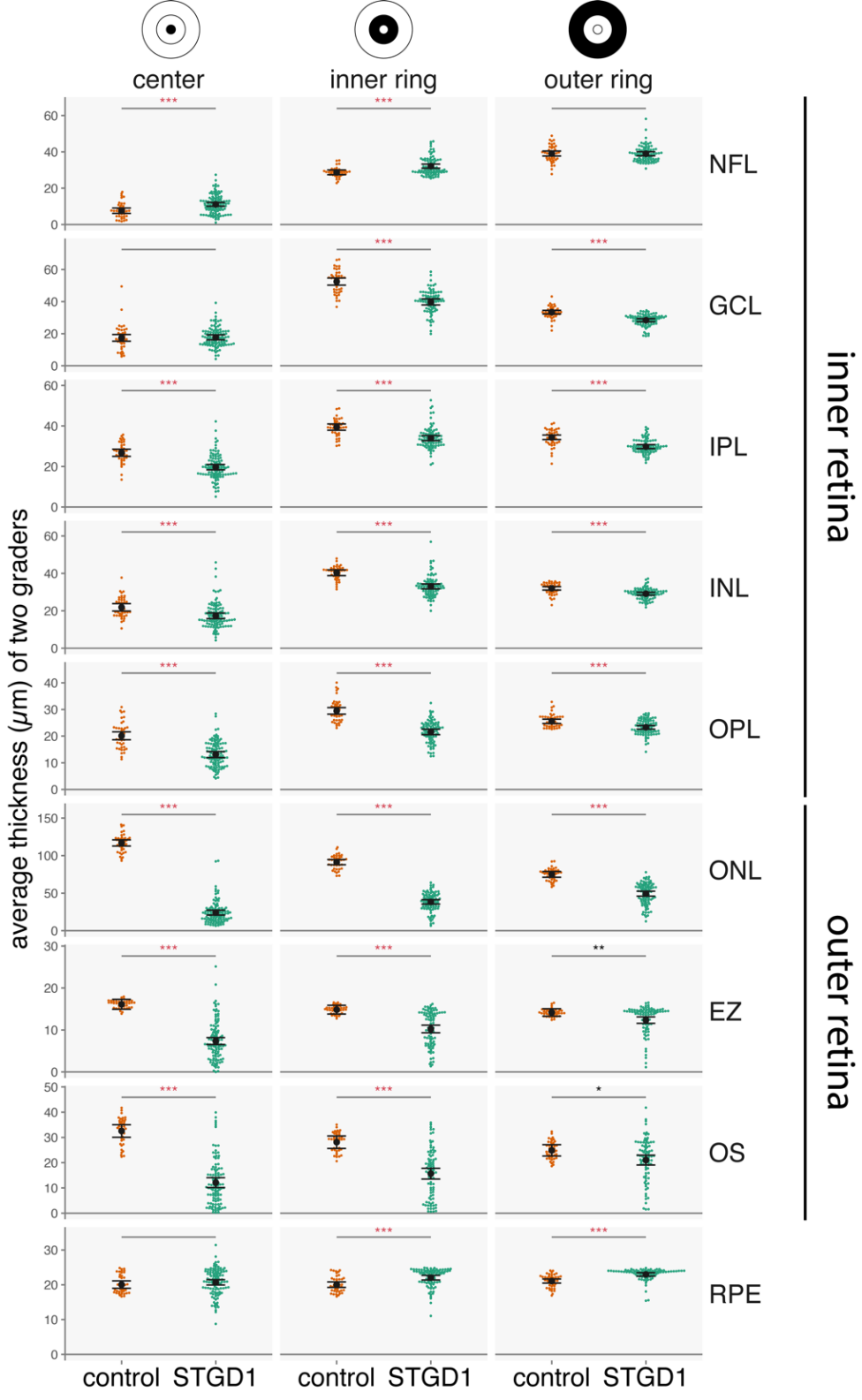
**Supplemental Figure 2.** Average baseline retinal sublayer thickness values for Stargardt (STGD1) versus normal controls.

Each point represents the thickness value in one eye (orange = normal control; green = STGD1). The magnitude of p-values for each estimate are indicated by asterisks (***: p < 0.001; **: p < 0.01; *: p < 0.05). Red indicates p < 0.0015 (Bonferroni corrected equivalent of p<0.05). NFL = nerve fiber layer; GCL = ganglion cell layer; IPL = inner plexiform layer; INL = inner nuclear layer; OPL = outer plexiform layer; ONL = outer nuclear layer; EZ = ellipsoid zone; OS = outer segment; RPE = retinal pigment epithelium. Generated from data using R (ver. 2.6.3; https://www.r-project.org/). ETDRS diagrams and annotations added in Adobe Illustrator (ver. 24.0.3; https://www.adobe.com/products/illustrator.html).

**Supplemental Figure 3.** Change in average thickness over time for Stargardt (STGD1) compared to normal controls.


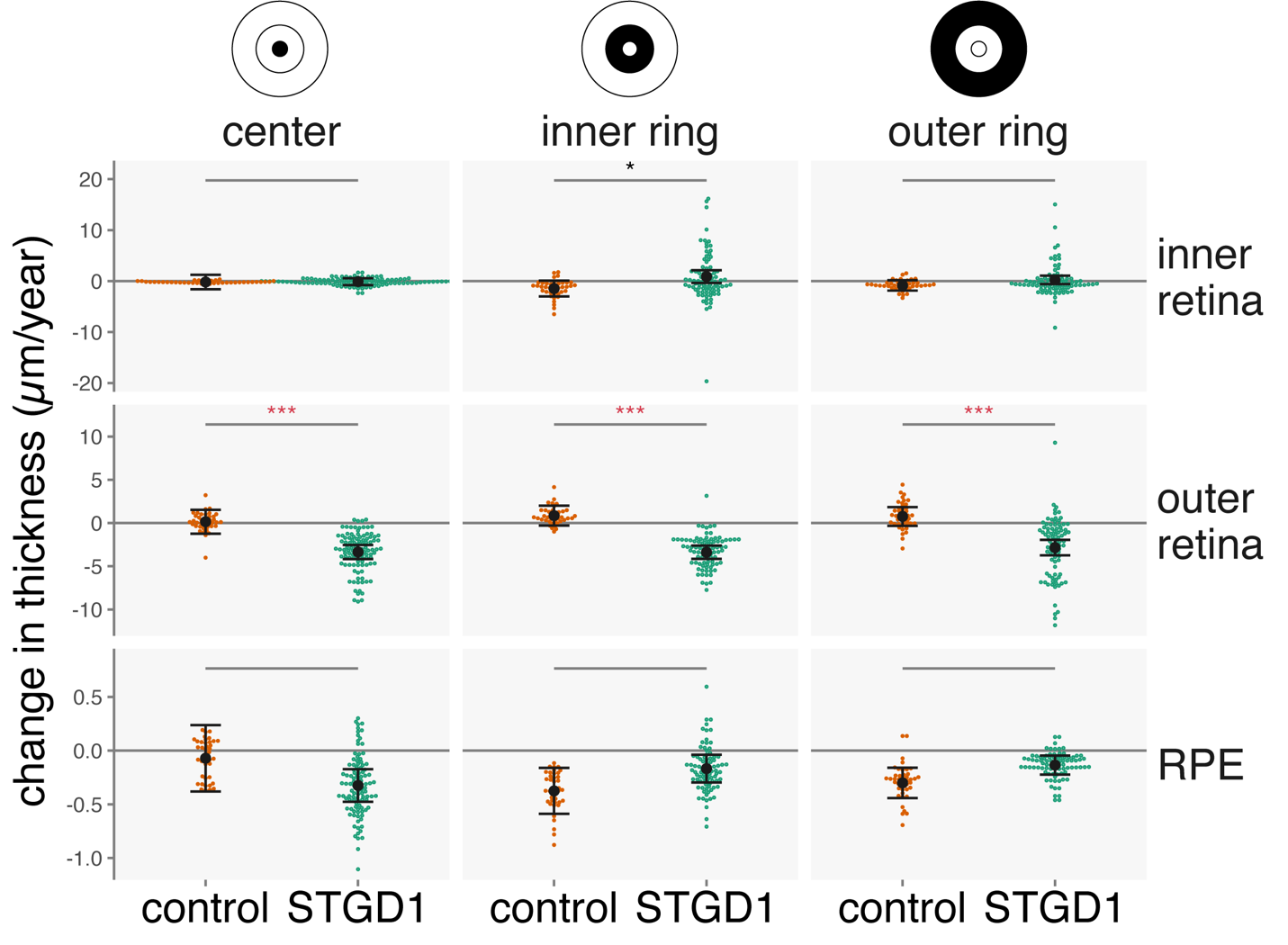


Each point represents the thickness value in one eye (orange = normal control; green = STGD1). The magnitude of p-values for each estimate are indicated by the number of asterisks (***: p < 0.001; **: p < 0.01; *: p < 0.05). Red indicates p < 0.0015 (Bonferroni corrected equivalent of p<0.05). Inner retina = nerve fiber layer to the outer plexiform layer. Outer retina = outer nuclear layer to the outer segments. RPE = retinal pigment epithelium. Generated from data using R (ver. 2.6.3; https://www.r-project.org/). ETDRS diagrams added in Adobe Illustrator (ver. 24.0.3; https://www.adobe.com/products/illustrator.html).

**Supplemental Figure 4.** Change in retinal sublayer thicknesses per year for Stargardt (STGD1) compared to normal controls.


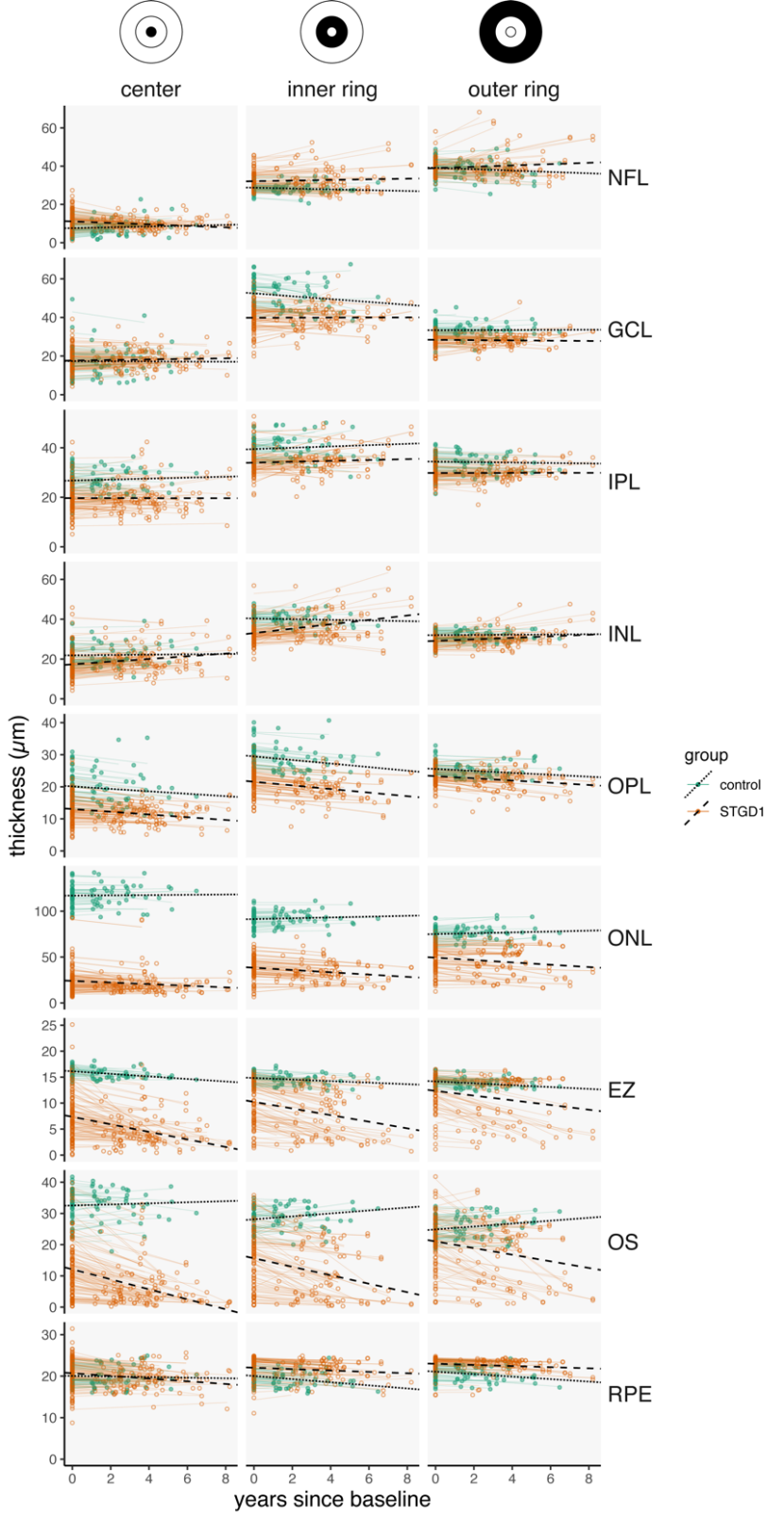


Thin colored lines indicate fits for individual eyes in the dataset (orange = normal control; green = STGD1). Thick black lines indicate group averages (solid line = control; dashed lines = ABCA4). NFL = nerve fiber layer; GCL = ganglion cell layer; IPL = inner plexiform layer; INL = inner nuclear layer; OPL = outer plexiform layer; ONL = outer nuclear layer; EZ = ellipsoid zone; OS = outer segment; RPE = retinal pigment epithelium. Generated from data using R (ver. 2.6.3; https://www.r-project.org/). ETDRS diagrams added in Adobe Illustrator (ver. 24.0.3; https://www.adobe.com/products/illustrator.html).

**Supplemental Figure 5.** Change in average thickness values over time for Stargardt (STGD1) compared to normal controls.


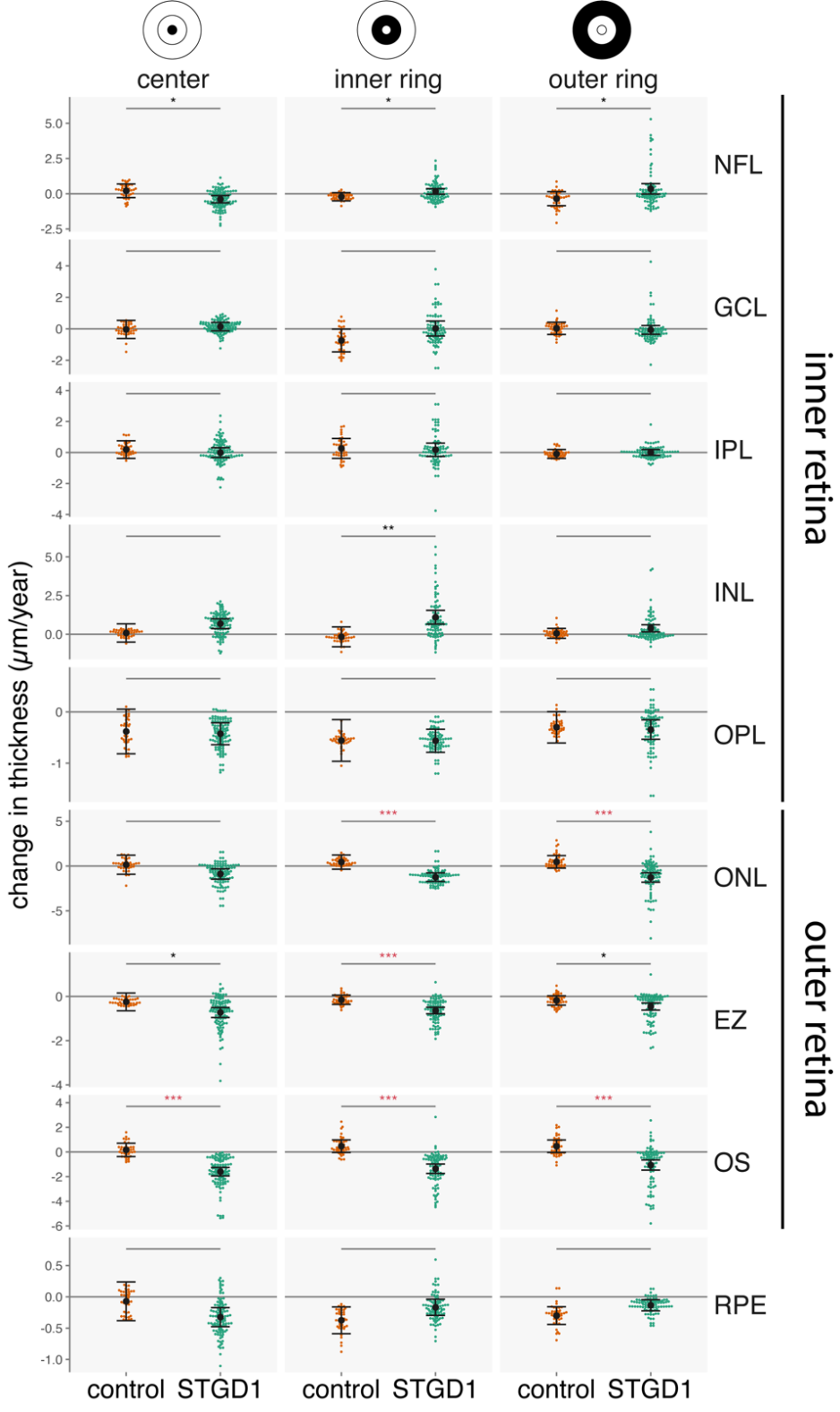


Each point represents the thickness value in one eye (orange = normal control; green = STGD1). The magnitude of p-values for each estimate are indicated by asterisks (***: p < 0.001; **: p < 0.01; *: p < 0.05). Red indicates p < 0.0015 (Bonferroni corrected equivalent of p<0.05). NFL = nerve fiber layer; GCL = ganglion cell layer; IPL = inner plexiform layer; INL = inner nuclear layer; OPL = outer plexiform layer; ONL = outer nuclear layer; EZ = ellipsoid zone; OS = outer segment; RPE = retinal pigment epithelium. Generated from data using R (ver. 2.6.3; https://www.r-project.org/). ETDRS diagrams and annotations added in Adobe Illustrator (ver. 24.0.3; https://www.adobe.com/products/illustrator.html).
